# Matrix Rigidity-Modulated Cardiovascular Organoid Formation from Embryoid Bodies

**DOI:** 10.1371/journal.pone.0094764

**Published:** 2014-04-14

**Authors:** Artem Shkumatov, Kwanghyun Baek, Hyunjoon Kong

**Affiliations:** 1 Department of Pathobiology, College of Veterinary Medicine, University of Illinois at Urbana-Champaign, Urbana, Illinois, United States of America; 2 Department of Materials Science and Engineering, University of Illinois at Urbana-Champaign, Urbana, Illinois, United States of America; 3 Department of Chemical and Biomolecular Engineering, Department of Pathobiology, Institute of Genomic Biology, University of Illinois at Urbana-Champaign, Urbana, Illinois, United States of America; Centro Cardiologico Monzino, Italy

## Abstract

Stem cell clusters, such as embryoid bodies (EBs) derived from embryonic stem cells, are extensively studied for creation of multicellular clusters and complex functional tissues. It is common to control phenotypes of ES cells with varying molecular compounds; however, there is still a need to improve the controllability of cell differentiation, and thus, the quality of created tissue. This study demonstrates a simple but effective strategy to promote formation of vascularized cardiac muscle - like tissue in EBs and form contracting cardiovascular organoids by modulating the stiffness of a cell adherent hydrogel. Using collagen-conjugated polyacrylamide hydrogels with controlled elastic moduli, we discovered that cellular organization in a form of vascularized cardiac muscle sheet was maximal on the gel with the stiffness similar to cardiac muscle. We envisage that the results of this study will greatly contribute to better understanding of emergent behavior of stem cells in developmental and regeneration process and will also expedite translation of EB studies to drug-screening device assembly and clinical treatments.

## Introduction

In a course of natural developmental and regeneration process, cells play central roles in forming complex tissues and organs via controlled proliferation, differentiation, and secretion of extracellular matrix molecules. For several decades, efforts have been made to better understand and further regulate these activities by orchestrating cell interactions with the extracellular matrix and neighboring cells. In these efforts, cells are often cultured to form a cluster [1], [2]. Such cell clusters can be further directed to form complex multicellular conglomerates *in vitro* towards generation of complex, three dimensional (3D) organoids useful to fundamental and applied bioscience studies.

Multicellular clusters are typically prepared by inducing aggregation between multiple types of tissue-specific cells suspended in culture medium or embedded in 3D gel matrices; however, this approach is often plagued by a limited cell source, poor controllability of spatial organization of cells, or a complex formulation of cell culture medium. For that purpose, embryoid bodies (EBs) derived from embryonic stem (ES) cells have been extensively studied, because pluripotent ES cells can unlimitedly generate desired tissue-specific cells via self-renewal and differentiation process. For example, a medium supplemented with certain soluble factors including retinoic acid and DMSO stimulated cardiomyogenic differentiation in EBs [3], [4]. Separately, a method was established to stimulate differentiation to Flk1 positive endothelial progenitor cells in EBs [5]. However, there is still a need to improve differentiation levels and finally create multicellular clusters with structure and functionality similar to tissues of interest.

According to recent studies, mechanical rigidity of a matrix, to which cells adhere, plays a significant role in regulating cellular phenotypes because cells are able to sense and respond to changes in their mechanical environment [6]. For instance, the differentiation of mesenchymal stem cells into a specific lineage is enhanced on a matrix designed to present stiffness similar to tissue of interest [7]. In addition, a cell adhesion substrate with the heart tissue-like stiffness (i.e., elastic modulus of 10 kPa) was shown to facilitate contraction/relaxation of cardiomyocytes, whereas scar-like stiff substrate prompted cells to lose their contractile activity [8]. Therefore, it is plausible that mechanical stiffness of a cell adhesion matrix may also modulate multidirectional differentiation of ES cells within EBs and further function of resulting organoids; however, few efforts have been made to systematically examine the role of matrix rigidity to date.

EBs are formed from ES cells grown in suspension on low adhesion culture dishes and present an intermediate stage for ES cell differentiation. ES cell differentiation inside EBs is a spontaneous process that is regulated by spatiotemporal arrangement of cells. The cells lining the EB surface belong to the primitive endoderm lineage, which gives rise to yolk sac in true embryos, whereas cells inside EBs represent populations of mesodermal, ectodermal and definitive endodermal origin. Unlike previously believed, EB differentiation is not random, but resembles early gastrulation events in embryos, and thus, resembles the natural process of development [9],[10]. Interestingly, an early attachment to the substrate is crucial for the prolonged embryo development *in vitro* [11]. Similarly, EB attachment to the substrate destroys radial symmetry, orients EBs and establishes bilateral symmetry that subsequently leads to the induction of mesoderm [12], and therefore, may promote the myocardial differentiation. However, substrates with high mechanical stiffness (1 GPa) were found to transform three-dimensional (3D) cell arrangement in EBs into a two-dimensional monolayer of cells on culture plastic [12]. Thus, we reasoned that a high mechanical stiffness of substrate and a relative paucity of cell-to-cell contacts may be inhibitory for myocardial and endothelial differentiation.

**Figure 1:**
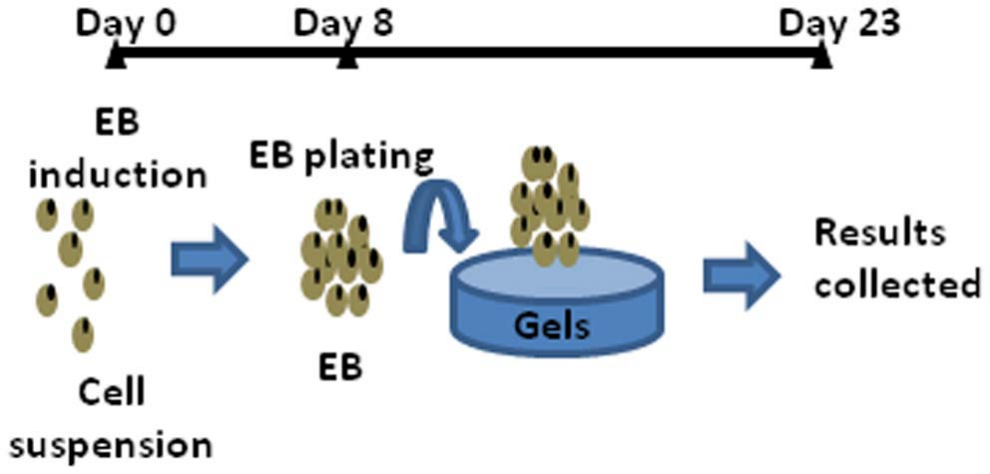
Schematic description of the experiment design. EBs were formed from the suspension of ES cells during the first 8 days. Then, EBs were cultured in suspension, on the pure collagen gel with *E* of 0.2 kPa or CCP gels with *E* of 6 and 40 kPa for additional 15 days.

In this study, we hypothesized that mechanical stiffness of a matrix, to which EBs adhere, would allow us to regulate cellular cardiomyogenic and endothelial differentiation within EBs and further tailor the cellular ultrastructure and contractile activity of resulting organoids. To examine this hypothesis, EBs derived from ES cells were cultured on a pure collagen gel and collagen-conjugated polyacrylamide (CCP) hydrogels with elastic moduli of approximately 0.2, 6, and 40 kPa (Fig. 1). After cell culture for 23 days, cellular differentiation levels and intercellular organization were examined using immunostaining and histological techniques. Additionally, the contractile activity of resulting cardiovascular organoids was examined by analyzing their beating frequency. Overall, this study will lead to a better understanding of the emergent behavior relevant to cardiovascular tissue development and regeneration.

## Materials and Methods

### Preparation of hydrogels

The collagen gel was prepared by reconstituting bovine type I collagen solution (PureCol, Advanced Biomatrix) with cold DMEM/F12 (Invitrogen). The final concentration of collagen was kept constant at 1.4 mg/ml. The resulting collagen gels were incubated at 37°C and 5% CO_2_ for two hours before plating EBs. Separately, to prepare CCP gels with different elastic moduli, 3 mg/ml collagen solution was first incubated with 50 mg/ml poly(ethylene glycol) *N*-hydroxysuccinimide ester (Acrylate PEG-NHS, Jenkem Technology) in ratio 10∶1 at 4°C for two hours. Then, the collagen bound with acrylate PEG-NHS was mixed with stock solutions of 40% acrylamide and 2% N,N′-methylenebis(acrylamide), and finally with 10% ammonium persulfate (Sigma) and 10% tetramethylethylenediamine (TEMED, Fluka). The molar ratio between 2% N,N′-methylenebis(acrylamide) and acrylamide was varied from 0.0017 to 0.017. The resulting gel was further incubated in phosphate buffered saline (PBS) at room temperature overnight before plating EBs on the gel. PBS was exchanged three times a day. All gels including pure collagen gels remained structural stable throughout the entire culture period. Microstructures of gel surfaces were analyzed with a scanning electron microscope (Hitachi 4800), using 1 kV acceleration voltage, 10 µA emission current and 4 mm working distance.

### Analysis of hydrogel stiffness

Mechanical stiffness of resulting CCP gels was evaluated by measuring elastic moduli of gels. After 24 hours of incubation in PBS, polyacrylamide gels were compressed at a rate 0.1 mm/min using a mechanical testing system (MTS Insight). The elastic modulus was calculated from the slope of the curve between stress and ratio of the deformed length to the un-deformed length. In the calculation, only the first 10% of strain region was considered. Three samples were measured per condition [13]. Separately, elastic modulus of the pure collagen gel was measured using a rheometer (Bohlin; CS-50). The collagen gel was prepared by reconstituting bovine type I collagen solution (PureCol, Advanced Biomatrix) with cold DMEM/F12 (Invitrogen) to a final concentration 1.4 mg/ml. The samples were prepared within the space between the walls of a rheometer's cup and bob. Then, the samples were oscillated at stress of 5 Pa to deform the gel within the linear viscoelastic region. The oscillation frequency was varied from 1 to 10 Hz.

### Undifferentiated embryonic stem cell culture

In all experiments, we used mouse embryonic stem cell line W4 (Taconic) with passage numbers between 17 and 22. The undifferentiated state was maintained by culturing cells in 85% DMEM/F12 (Mediatech, CellGro^R^) and 15% Knockout Serum Replacement (Invitrogen) on mitomycin-treated mouse embryonic fibroblast feeder cell monolayer [14]. The medium was also supplemented with 0.1 mM 2-Mercaptoethanol (Invitrogen), 1∶100 GlutaMAX™ (Invitrogen), and 1000 U/ml mouse leukemia inhibitory factor (Sigma). ES cells were re-plated on gelatin (Sigma) – coated dishes 3 days before harvesting them to prepare EBs.

### Embryoid body (EB) culture

Undifferentiated ES cell colonies were dissociated by incubating them in a 0.05% trypsin solution (Mediatech). The dissociated cells were placed on polystyrene-based six-well cell culture plates (Becton Dickinson) and incubated in differentiation medium with or without serum. Differentiation medium was composed of 90% DMEM/F12 (Mediatech), 10% fetal bovine serum (FBS, Invitrogen), 0.1 mM 2-Mercaptoethanol (Invitrogen), 1∶100 GlutaMAX™ (Invitrogen), 100 U/ml penicillin, and 100 µg/ml streptomycin. In serum-free medium, FBS was substituted with Knockout Serum Replacement (Invitrogen). The six-well culture plates were placed on a rotary shaker (Heidolph Rotamax 120) at 30 rpm and incubated at 37°C and in 5% CO_2_ without interruptions. After four days, EBs were transferred into 25 cm^2^ flasks treated with 2.3% agar (Sigma) and cultured for another four days. On 8^th^ day, all EBs were split in equal numbers into four groups: EBs cultured in suspension, EBs plated on a collagen gel with a shear modulus of 0.2 kPa, and on CCP gels with shear moduli of 6 and 40 kPa (Fig. 1). The control non-adherent “suspended” EBs were cultured on collagen-free polyacrylamide gels with an elastic modulus of 6 kPa. All analyses were performed on 23^rd^ day of observation. The beating frequency was measured with a timer while observing individual EBs under microscope.

### Histological analysis

After EBs had been fixed in 10% buffered formalin for four hours and embedded in paraffin, they were sectioned into 3 µm thick sections. The sections were stained with Hematoxylin and Eosin (H&E), and photographed with a NanoZoomer Slider Scanner/Digital Pathology System (Hamamatsu). Areas filled with uniform eosinophilic cellular debris, with lost differential staining of nuclear and cellular details, were recognized as areas of necrosis. Fraction of necrotic cells in histological section was quantified by counting numbers of positively stained pixels using NIH ImageJ software.

### Total RNA purification

EBs were briefly treated with a 0.025% solution of collagenase 1 (Sigma) for detachment from the substrate and washed once with PBS. Total RNA was extracted using an RNeasy Mini Kit (Qiagen) following the manufacturer's instructions. The tissue was disrupted and homogenized by vortexing it using a syringe with 20 G needle. The concentration of RNA was measured using a spectrophotometer (ND-1000, NanoDrop); and the quality of RNA was confirmed with 2100 Bioanalyzer (Agilent).

### Real time PCR

cDNA was synthesized from equal amounts of total RNA using random hexamer primers and the SuperScript III First-Strand Synthesis System (Invitrogen) as per manufacturer's instruction. The amplification of cDNA was performed on the Applied Biosystems 7900HT Fast Real-Time PCR System. Molecular Probes Assays (Mm00473657_m1 and Mm01290256_m1, Invitrogen) were used for amplification of Actn2 and Tnnt2 mRNA, respectively. The reaction was carried out at 50 °C for 2 min, 95 °C for 10 min, then at 95 °C for 15 sec and at 60 °C 1 min for 40 cycles. For each sample, PCRs were performed in duplicate or triplicate. The cDNA levels were determined using the standard curve of cycle thresholds. The results were normalized against the mouse GAPDH cDNA (Molecular Probes Assay ID: Mm99999915_g1).

### Immunohistochemical imaging

EBs were fixed in 10% buffered formalin for one hour, and incubated in 0.5% Triton X in PBS at 4 °C overnight. Then, EBs were blocked in PBS with 3% bovine serum albumin for two hours and stained overnight with primary antibodies against CD31 (Abcam, ab28364) and against sarcomeric α-actinin (Abcam). EBs were washed three times with PBS before exposure to secondary antibodies: goat anti-rabbit IgG (H+L) (DyLight 488, ThermoScientific) and polyclonal Alexa Fluor 568 Goat anti-mouse IgG (H+L) (Abcam). After overnight incubation at 4 °C, EBs were washed three times and mounted on glass slides with mounting medium Prolong^R^ Gold Antifade Reagent (Invitrogen). Mounted EBs were flattened into disks with thickness 20–30 µm on the slides. Finally, EBs were imaged using a laser scanning confocal microscope (Zeiss LSM 700). Specifically, sarcomeric α-actinin was imaged by exciting samples at 555 nm and collecting emission over 560 nm. CD31 was imaged by exciting samples at 488 nm and collecting emission ranging from 488 nm to 550 nm. Positively stained area in individual EBs was quantified using NIH ImageJ software.

### Flow cytometry

EBs were broken down into a single cell suspension using Liberase Blendzyme 2 (0.1 mg/ml, Roche Diagnostics) and fixed in 4% neutral-buffered formalin. After permeabilization with a 0.1% Triton-X solution for 5 min, EBs were double-stained with primary antibodies against CD31 (Abcam, ab28364) and against sarcomeric α-actinin (Abcam). Next, we applied the following secondary antibodies: goat anti-rabbit IgG (H+L) (DyLight 488, ThermoScientific) and polyclonal Alexa Fluor 568 Goat anti-mouse IgG (H+L) (Abcam). FACS analysis was performed with the flow cytometry analyzer (**BD FACSAria** II).

### Cell cycle analysis

Proliferating cells in EBs were labeled with Click-iT EdU Alexa Fluor 488 Imaging Kit following manufacturer's instruction. In brief, EBs were incubated with EdU for 24 hours and fixed on days 15 and 23. At the same time, cardiomyocytes within EBs were stained for sarcomeric α-actinin as described above. Cell nuclei were marked with Hoechst 33342 in the final concentration 5 µg/ml. Again, EBs were imaged using a laser scanning confocal microscope (LSM 700, Zeiss), with at least 1000 cells counted for each group. The cells incorporating EdU contained nuclei stained in green color. The nuclei stained only by Hoechst (blue) were considered negative. The cells with green nuclei and red cytoplasm were considered double positive for the EdU incorporation and cardiomyoblast marker SAA.

### Morphometric analysis

NIH ImageJ software was used for all morphometric analyses of EBs. EB diameter was measured with images captured using an inverted microscope (DMI 4000B, Leica) equipped with a digital camera ORCA-ER (Hamamatsu Photonics). The size of embryoid bodies was measured by drawing a line along the short axis of an EB. Such measurement characterizes the lesser diameter of an EB.

### Statistical analysis

At least, three repeats were done for each experimental group; and 50 EBs or more were analyzed for each condition unless indicated otherwise. Statistical significance was determined via Student's *t* test, where p<0.05. The data is presented with mean ± SE unless indicated otherwise.

## Results

### Assembly of EB-adherent hydrogels with controlled elastic moduli

Collagen-based hydrogels capable of inducing EB adhesion on their surfaces were assembled to present controlled elastic moduli while keeping collagen density in the gels constant. Gels with an elastic modulus (*E*) of 0.2 kPa were prepared by changing the pH of precursor collagen solution, with collagen concentration 1.4 mg/ml, to 7.3 (Table 1). *E* of the gel was further increased to 6 and 40 kPa by introducing mixture of acrylamide, acrylated PEG-NHS and varying amounts of bis-acrylamide into 1.4 mg/ml collagen solution and activating *in situ* polymerization and cross-linking reactions (Fig. S1). The acrylated PEG-NHS chemically linked collagen to polyacrylamide. Increasing elastic modulus of the CCP gel resulted in a decrease of the swelling ratio (Table 1). All gel surfaces presented interconnected collagen fibers, as confirmed with SEM images (Fig. S2). Additionally, the gels remained structurally stable throughout entire cell culture period, without showing any deformation or structural disintegration.

**Table 1:** Composition and properties of hydrogels.

Hydrogel	M	E (kPa)	Q
Pure collagen gel	N/A	0.2	80.9±2.3
CCP gel	0.0017	6.0±0.8	47.1±1.9
CCP gel	0.017	40.0±1.1	19.5±1.7

M: Molar ratio of N,N′-methylenebis(acrylamide) to acrylamide; E: Elastic modulus, Q: Degree of swelling.

### Effects of matrix stiffness on growth and microstructure of EBs

**Figure 2:**
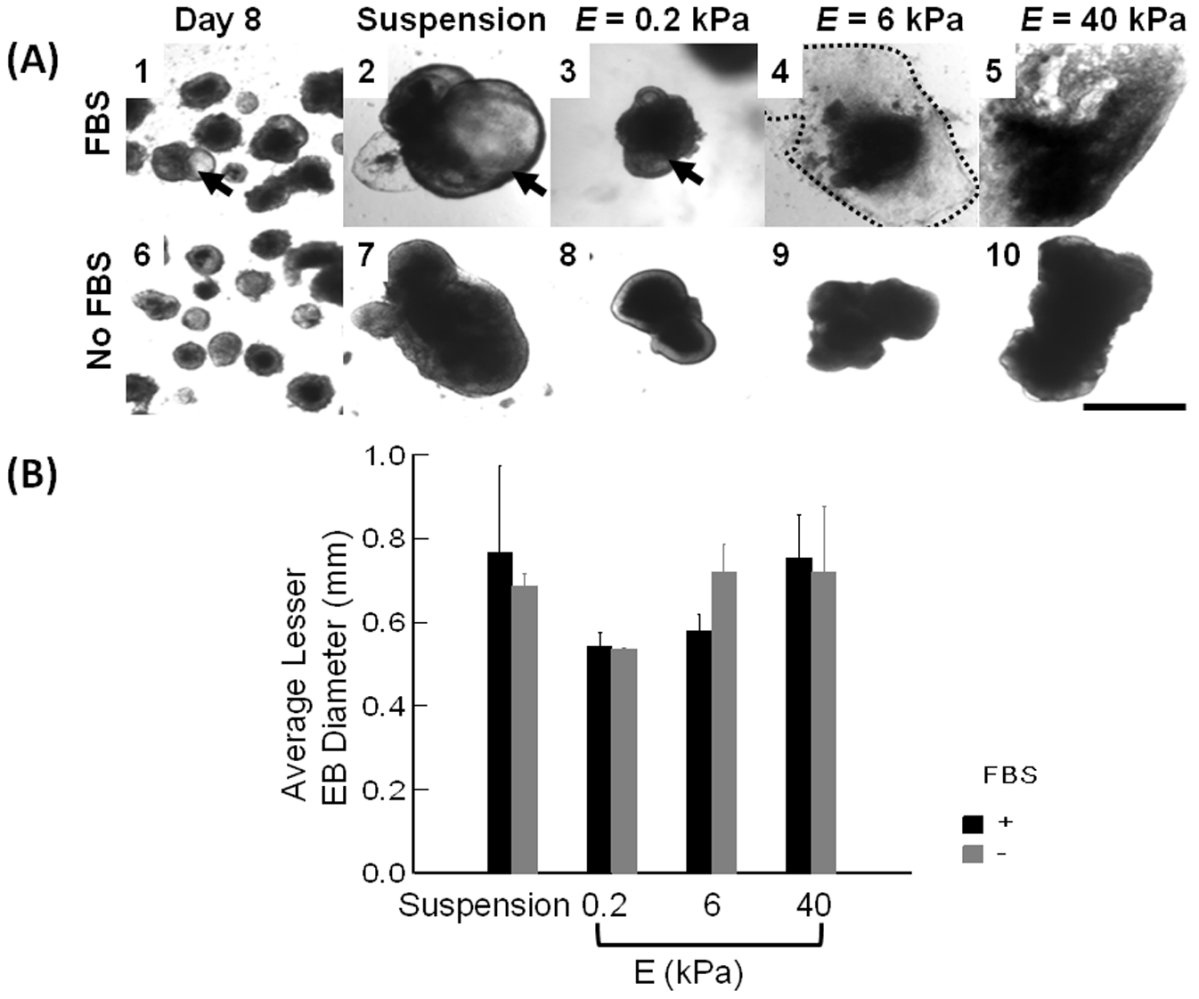
Size analysis of EBs cultured in suspended state or on hydrogels with controlled elastic moduli. (A) Bright field images of EBs. (A-1 and A-6) EBs formed by culturing ES cells in suspended state for 8 days. (A-2 and A-7) EBs cultured in suspended state for additional 15 days. (A-3 and A-8) EBs cultured on the pure collagen gel with *E* of 0.2 kPa, (A-4 and A-9) EBs cultured on the CCP gel with *E* of 6 kPa, and (A-5 and A-10) EBs cultured on the CCP gel with *E* of 40 kPa. Images in the first and second rows represent EBs cultured in medium supplemented with 10% FBS and that without FBS, respectively. Arrows in A-1 to A-3 indicate cystic EBs. The scale bar represents 1 mm. (B) The quantified analysis of average diameters of EBs cultured in suspended state or on collagen-based hydrogels of controlled *E*. Black bars represent average diameter of EBs cultured in the medium supplemented with 10% FBS, and grey bars represent EBs cultured without FBS. Values and error bars represent the mean and the standard error of at least 50 EBs, respectively. No statistical significance was found between conditions.

EBs were induced on Day 0 by culturing ES cells on low adhesive dishes using rotary shaker. Rotary EB culture provided us with large numbers of EBs with circular shape and uniform size. On Day 8, an average diameter of EBs was 319±19 µm (not shown). About 24–48 hours after plating, EBs adhered to the collagen-conjugated gels and were stably immobilized on the gel surface until the end of the experiment. By contrast, EBs plated on collagen-free gels did not adhere to the substrate and maintained three-dimensional spherical shape (Fig. 2A-2). The continued culture of EBs in a suspended state or on the gels with different elastic moduli for a total of 23 days led to a three-fold increase of the average diameter compared to 8 day old EBs (not shown). There was no statistically significant difference in the average diameter between EBs cultured with and without FBS (Fig. 2B).

**Figure 3:**
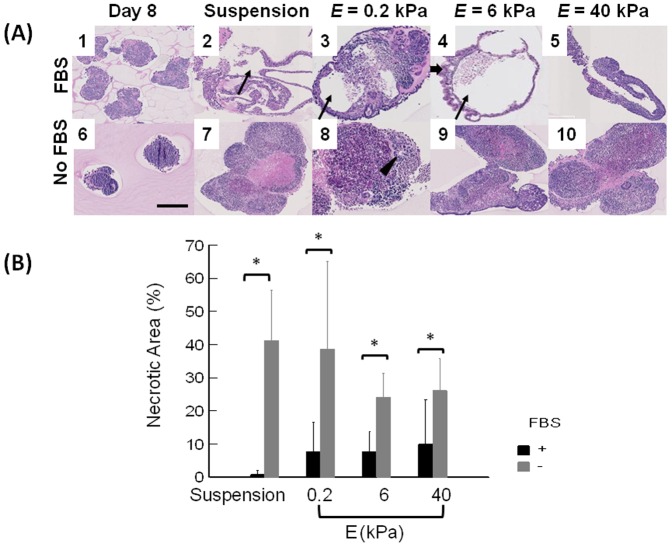
Histological analysis of EBs cultured in suspended state or on hydrogels with controlled elastic moduli. Cross-sections of EBs were stained with Hematoxylin & Eosin. (A) (A-1 and A-6) EBs formed by culturing ES cells in suspended state for 8 days. (A-2 and A-7) EBs cultured in suspended state for additional 15 days. (A-3 and A-8) EBs cultured on the pure collagen gel with *E* of 0.2 kPa, (A-4 and A-9) EBs cultured on the CCP gel with *E* of 6 kPa, and (A-5 and A-10) EBs cultured on the CCP gel with *E* of 40 kPa. Images on the first and second rows represent EBs cultured in medium supplemented with 10% FBS and that without FBS, respectively. Thin arrows in A-2, 3, and 4 indicate cystic EBs. The thick arrow in A-4 indicates columnar epithelium, and the arrowhead in A-8 indicates a neuroectodermal rosette. Scale bar represents 250 µm. (B) Quantified necrotic area percentage of EBs cultured in suspension and on hydrogels with *E* of 0.2, 6, and 40 kPa. Black bars represent average diameter of EBs cultured in media supplemented with 10% FBS and grey bars do those cultured without FBS. The difference of values for EBs cultured in the medium supplemented with 10% FBS (black bar) and free of FBS (grey bar) is statistically significant for all four different conditions (*p<0.05). Values and error bars represent the mean and the standard deviation of at least 10 EBs, respectively.

The spatial organization and viability of cells within EBs was further analyzed by staining cross-sections of EBs with H & E. EBs cultured in FBS-free medium were composed of tightly packed cells (Images in the second row of Fig. 3A) independent of elastic modulus of the gel; and EB cores contained large necrotic areas largely filled by cells, with lost differential staining of nuclei and cytoplasm. In contrast, EBs cultured in FBS-supplemented medium often contained large cavities (cysts) in their cores (Images in the top row of Fig. 3A). According to quantitative analysis, there was almost ten-fold increase of the necrotic area within EBs cultured in the FBS-free medium (Fig. 3B). Additionally, EBs cultured in FBS-supplemented medium displayed higher levels of cell organization, such as palisades of epithelial cells, epithelium lined cavities, and blood vessels that are major components of different embryonic lineages (Fig. S3). Therefore, only EBs cultured in FBS-supplemented medium were used for analysis of their co-differentiation in this study.

### Effects of matrix stiffness on cardiomyogenic and endothelial differentiation and subsequent cardiovascular tissue-like structure within EBs

**Figure 4:**
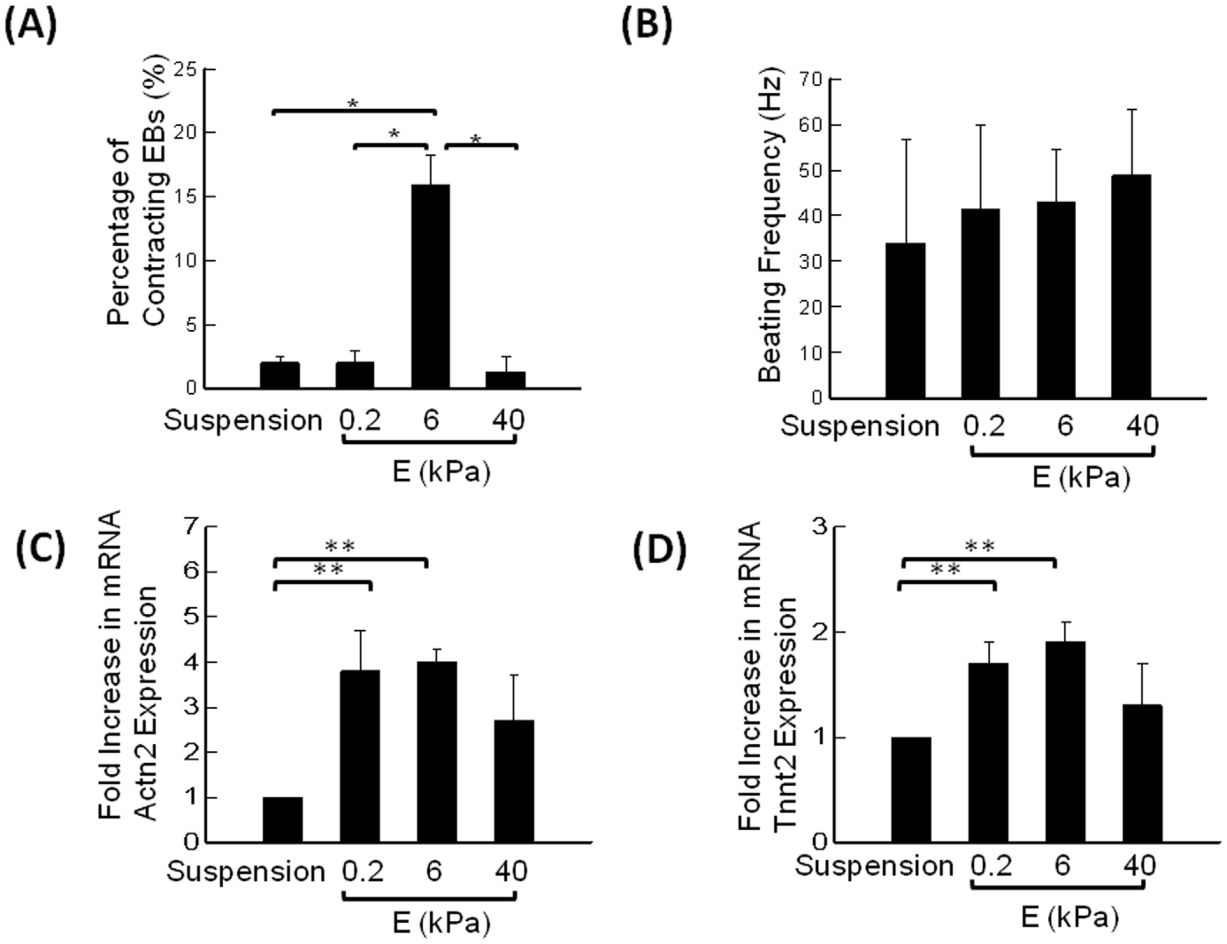
Analysis of stiffness-modulated contraction in EBs. (A) Percentage of contracting EBs. The difference of the values between EBs cultured on the gel with *E* of 6 kPa and other three conditions is statistically significant (*p<0.05). Values and error bars represent the mean and the standard error of at least 100 EBs, respectively. (B) Frequency of EB contractions. Values and error bars represent the mean and the standard deviation of at least 5 EBs, respectively. (C) Effects of *E* of the hydrogel on the sarcomeric α-actinin (Actn2) mRNA expression. (D) Effects of *E* of the hydrogel on cardiac troponin T type 2 (Tnnt2) mRNA expression. Values and error bars represent the mean and the standard error. In (C) and (D), * indicate statistical significance of difference between conditions (*p<0.05).

The EBs cultured in FBS-supplemented medium exhibited contractile activity from the second week of culture. Interestingly, the percentage of EBs with contractility was seven-fold higher on the gel with *E* of 6 kPa than other three conditions, including EBs cultured in suspended state and those adhered to gels with *E* of 0.2 and 40 kPa (Fig. 4A). By contrast, the difference in beating frequency found between four groups was not statistically significant (Fig. 4B).

Cardiomyogenic differentiation level was first evaluated by quantifying mRNA levels of Actn2 and Tnnt2 using real – time PCR. Actn2 and Tnnt2 genes encode sarcomeric α-actinin (SAA) and cardiac troponin T type 2, respectively, with both being markers of advanced cardiomyogenic differentiation in ES cells [15]. The expression of SAA and cardiac troponin was significantly elevated in EBs adhered to hydrogels compared to the EBs cultured in suspension for an entire period (Fig. 4C and D). There was a slight increase in expression of both genes on the gel with *E* of 6 kPa; however, this finding was not statistically significant as measured by qRT-PCR (Fig. 4C and D). Such difference between mRNA levels and the percentages of contractile EBs is explained by the fact that mRNA levels include cardiomyocytes that do not contract.

**Figure 5:**
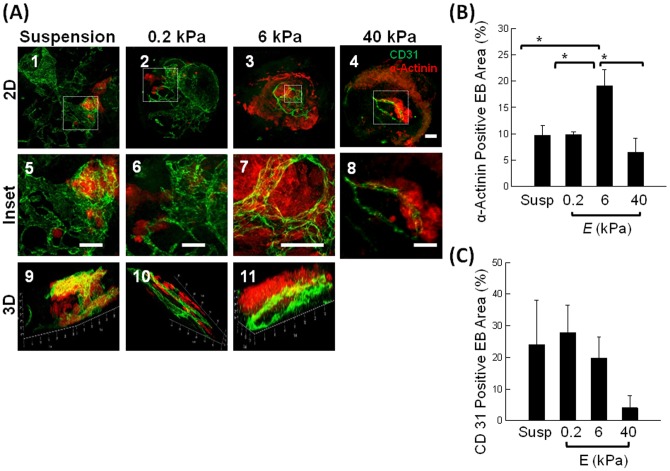
Immunohistochemical analysis of cardiomyogenic and endothelial differentiation within EBs. (A) Fluorescent images of EBs stained for sarcomeric α-actinin (red) and CD31 (green). (A-1, 5, & 9) EBs cultured in suspended state for 23 days. (A-2, 6 & 10) EBs cultured on the pure collagen gel with *E* of 0.2 kPa. (A-3, 7, & 11) EBs cultured on the CCP gel with *E* of 6 kPa. (A-4 & 8) EBs cultured on the CCP gel with *E* of 40 kPa. Scale bar represents 200 µm. Images on the second row are magnified views of those on the first row. Images on the third row are three-dimensional confocal images of EBs. (B) Percentage of the EB area positively stained by antibodies to sarcomeric α-actinin. The difference of the values between EBs cultured on the gel with *E* of 6 kPa and other three conditions is statistically significant (*p<0.05). (C) Percentage of EB area positively stained with an antibody to CD31. The difference of the values between EBs cultured on the gel with *E* of 6 and 40 kPa is statistically not significant (*p>0.1). Values and error bars represent the mean and the standard error of at least 20 EBs, respectively.

**Figure 6:**
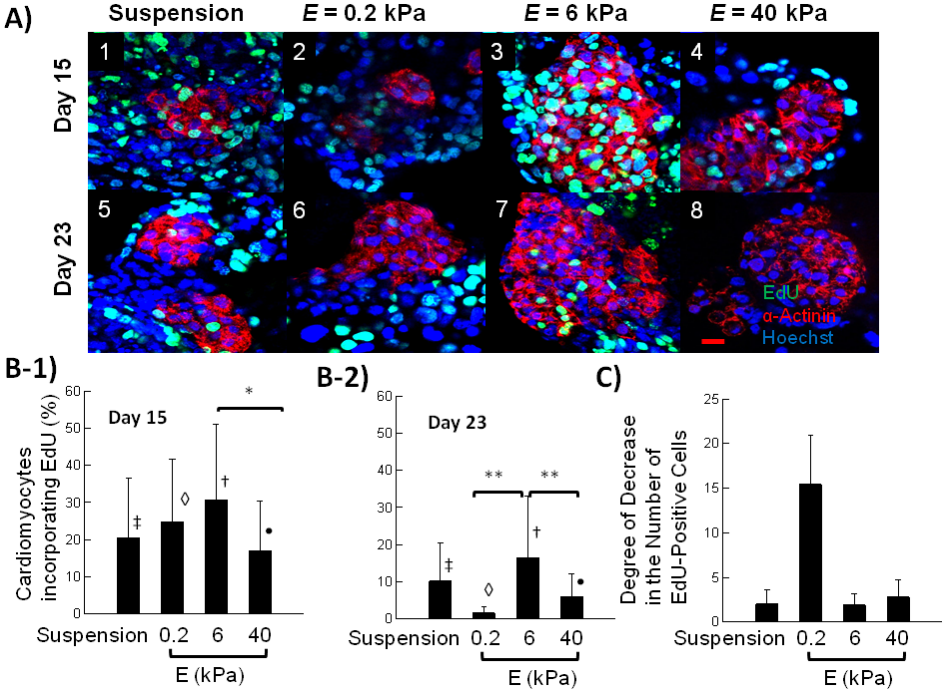
Cell cycle analysis of cardiomyoblasts using EdU incorporation. (A) Fluorescent images of sarcomeric α-actinin positive cells (red) incorporating EdU (green). Blue color represents cell nuclei stained by Hoechst 33342. Images represent EBs cultured in suspended state (A-1 and A-5) and on hydrogels with *E* of 0.2 (A-2 and A-5), 6 (A-3 and A-5), and 40 kPa (A-4 and A-5). Scale bar represents 20 µm. Images on the first row were taken on Day 15, and those on the second row were taken on Day 23. (B) Quantified percentage of cardiomyoblasts incorporating EdU on Day 15 (B-1) and 23 (B-2). In (B-1), the difference of values between EBs cultured on the gel with *E* of 6 kPa and 40 kPa was statistically significant (*p<0.05). In (B-2), the difference of values between EBs cultured on the gel with *E* of 6 kPa and EBs on the gels with *E* of 0.2 and 40 kPa was statistically significant (**p<0.05). Symbols ‡, ◊, †, •, *, ** and brackets indicate statistically significant groups (p<0.05). (C) The degree of decrease in the percentage of cardiomyoblasts incorporating EdU between Day 15 and Day 23.

The cardiomyogenic and endothelial differentiation levels and reorganization of EBs were further analyzed with immunostaining for sarcomeric α-actinin (SAA) and CD31, respectively (Fig. 5). It is well reported that EBs do not produce skeletal muscles during differentiation, due to the absence of signals from the neural tube and notochord crucial for patterning of paraxial mesoderm [16]. Therefore, cells positively stained for SAA were considered cardiomyocytes. Our current staining protocol allowed visualization of all cells within EBs after flattening into disks with 20–30 µm thickness and the morphometric quantification of different cell types by circumscribing the positively stained areas of EBs. Confocal images of EBs cultured on the gel with *E* of 6 kPa displayed striated SAA-positive cardiomyocyte sheets with distinct CD31-positive blood vessel-like hollow tubes (Fig. 5A - 3, 7, & 11). In contrast, EBs cultured on the gel with *E* of 0.2 kPa showed large fractions of CD31-positve blood vessel-like hollow tubes and small islands of SAA-positive cardiomyocytes (Fig. 5A-2, 6, & 10), similar to EBs cultured in a suspended state (Fig. 5A-1, 5, & 9). EBs cultured on the gel with *E* of 40 kPa exhibited reduced formation of striated SAA-positive sheets (Fig. 5A-4 & 8).

According to quantitative image analysis, EBs cultured on the gel with *E* of 6 kPa displayed almost a two-fold increase in the percentage of SAA-positive staining, as compared to EBs suspended in medium or EBs plated on gels with *E* of 0.2 and 40 kPa (Fig. 5B). The average percentage of CD31-positve staining that represents endothelial differentiation level was inversely related to *E* of the gel (Fig. 5C); however, the difference between the conditions was not statistically significant.

Additionally, we analyzed the numbers of cells positive for SAA and CD31 by FACS (Fig. S4). The maximum differentiation efficiency into cardiomyocytes was similarly achieved on the gel with *E* of 6 kPa. The percentage of SAA-positive cells was 6.9, 1.4 and 1.2 times higher than in EBs cultured in suspension, and on the gels with *E* of 0.2 and 40 kPa, respectively. SAA+ CD31+ double – positive cells were also considered to be cardiomyocytes because SAA-positive cardiomyocytes form from endothelial progenitor cells and mesangioblasts [17], [18]. Interestingly, double positive cells were significantly increased on the gel with *E* of 6 kPa with 7.5-fold, 5-fold and 1.9-fold differences compared to EBs cultured in suspension and on the gels with *E* of 0.2 and 40 kPa, respectively. The percentage of double positive cells on the gel with *E* of 6 kPa was almost half of the number of cells positive for SAA only.

### Analysis of cell proliferation within EBs

The fraction of cardiomyogenic progenitor cells in EBs can be increased by one of two mechanism: (1) an increase in cardiomyogenic differentiation level, and (2) an increase in proliferation of cells already expressing cardiac markers, such as SAA and cardiac troponin. In order to address underlying mechanism, we assessed effects of gel stiffness on the proportion of cardiomyoblasts undergoing cell division cycle. EBs were incubated with 5-ethynyl-2′-deoxyuridine (EdU), which is a nucleoside analog of thymidine incorporated into DNA during active cell division. Cardiomyoblasts were identified by the positive staining for SAA and incorporation of EdU. According to the EdU assay performed on Days 15 and 23, EBs cultured on the gel with *E* of 6 kPa contained the highest percentage of EdU-positive cardiomyoblasts compared to other three conditions (Fig. 6). Specifically, on Day 23, the percentage of proliferating cells within EBs cultured on the gel with *E* of 6 kPa was almost one-order of magnitude higher than in EBs cultured on the gel with *E* of 0.2 kPa and also 2.5 times higher than in EBs on the gel with *E* of 40 kPa (Fig. 6 B-2).

Interestingly, EBs cultured on the gel with *E* of 0.2 kPa experienced the greatest drop in the percentages of cells undergoing cell division between Day 15 and 23: approximately 15-fold decrease (Fig. 6C). On Day 23, there was only a minimal percentage of proliferating cardiomyoblasts. In contrast, EBs cultured on the gel with *E* of 6 kPa showed only a 2-fold decrease in the number of dividing cells.

Additionally, we examined EdU incorporation in non-cardiomyocytes - cells negative for SAA. There was no statistical difference of the percentages of dividing cells on Day 15, with values close to 50% for all groups (Fig.S5 A-1). However, on Day 23 the percentage of EdU-positive cells on the gel with E of 0.2 kPa was more than 2-fold smaller than in EBs in suspension and those on a gel with *E* of 6 kPa (Fig.S5 A-2). Moreover, on the gel with *E* of 0.2 kPa, numbers of proliferating cells were reduced by more than 3 times between Days 15 and 23 of observation (Fig.S5 B).”

## Discussion

This study demonstrates for the first time that stiffness of cell-adherent matrix can modulate cardiomyogenic and endothelial differentiation of EBs, and further tailor the internal structure of cardiovascular organoids. We used rotary culture to produce large numbers of EBs with uniform size and circular shape as was reported previously [1]. EBs attached to the CCP gels exhibited active growth in a FBS-supplemented medium with minimal necrosis. Moreover, FBS-supplemented medium induced formation of cystic EBs that contained central cavities. Attached EBs also underwent cardiomyogenic and endothelial differentiation, more actively than those cultured in a suspended state. Additionally, their differentiation levels were dependent on *E* of the gel in a dissimilar manner. The percentage of contractile EBs, the level of cardiomyogenic differentiation, and frequency of cardiomyoblasts division were maximal when EBs were cultured on the gel with *E* of 6 kPa. Therefore, the culture of EBs on the gel with *E* of 6 kPa resulted in contractile EBs incorporating striated cardiac muscle and branched endothelial tubes.

Results of this study manifest that ES cells within EBs can recognize the difference in substrate stiffness, where they actively interact in a three-dimensional environment. As evidenced by a minimal dependency of cardiomyogenic differentiation on stiffness of non – adhesive collagen-free gels, the insoluble signal of matrix rigidity should be transmitted through specific bonds between cellular adhesion molecules (for example, collagen binding α1β1 and α2β1 integrins [19]) and collagen. Therefore, it is likely the hydrogel with stiffness of cardiac muscle (i.e., *E* of 6–10 kPa) biomechanically stimulated ES cells to activate signaling related to cardiomyogenic differentiation or proliferation, as confirmed by an increased population of α-actinin positive cardiomyocytes. This response of cell aggregates to the gel stiffness is similar to findings previously made on single cells cultured on gels with varying stiffness [20]. Additionally, this gel stiffness must be proper to stimulate proliferation of cardiomyoblasts within EBs, as demonstrated by an increased number of EdU-incorporating cells. We thus suggest that cardiomyoblast proliferation and subsequent differentiation to cardiomyocytes generate the largest percentage of EBs with contractile activity on the hydrogel with *E* of 6 kPa.

In contrast, limited cardiomyogenic differentiation of ES cells cultured on the hydrogel with stiffness of brain or fat tissue (i.e., *E* of 0.2 kPa) indicates that matrix is too soft to transmit an insoluble mechanical signal involved in cardiomyogenic differentiation into cells. Additionally, the limited proliferation of cardiomyoblasts would be another limiting factor towards formation of contracting EBs on such matrix. This is corroborated by an almost 8-fold drop in numbers of EdU-positive cells between Days 15 and 23. On the softer matrix, cells tend to associate with themselves rather than migrate on the matrix, thus likely precluding further multiplication via cell contact inhibition. It is unlikely that the reduced cell differentiation is caused by the difference in the collagen density of the pure collagen gel, because surface density of collagen was adjusted to be equivalent between all three conditions. Therefore, the difference in the cardiomyogenic differentiation level should be largely attributed to the difference of gel stiffness.

We also suggest that decreased cardiomyogenic differentiation in EBs cultured on a gel with stiffness of non-mineralized bone matrix (i.e., *E* = 40 kPa) should result from reduction in cell-to-cell contacts due to active cell migration out of EBs and disruption of 3D environment as we observed. Similar to previous studies, stiffer matrix likely activated cellular integrin expression, thus reducing intercellular adhesion within 3D EBs. These changes in the cellular organization should result in down-regulation of ES cell differentiation to cardiomyocytes. We also interpret that reduced proliferation rates of cardiomyoblasts on stiff gels recapitulate natural decrease of proliferation by neonatal cardiomyocytes. It is known that the rise in myocardial stiffness during embryological development [21] coincides with the rapid exit from the cell cycle by neonatal cardiomyocytes. Between embryonic day 14.5 and neonatal day 7, the percentage of actively proliferating cardiomyoblasts falls from 23% to 1% [22]. This marked reduction in proliferative capacity of cardiomyoblasts *in vivo* is surprisingly in line with our findings in the cell cycle altered by the gel stiffness *in vitro*. Additionally, the minimal difference in the EB size between conditions, as shown in Figure 2B, suggests that an increase in one cell population compensates for a reduction in cell numbers of other cell populations [23].

The results of flow cytometry further corroborated the morphological analysis, which indicated the importance of substrate stiffness in regulating cardiomyogenic differentiation of EBs. One interesting finding is that over 30% of cells positive for SAA were also positive for CD31 on the gels with *E* of 6 kPa. This result implies that EBs cultured on the intermediate stiff gel contained large number of incompletely differentiated cardiomyocytes likely formed through trans-differentiation [18].

Gene expression analysis performed by qRT-PCR was in general supportive of the morphometric analysis. It was reported that gene expression analysis of EBs is a very challenging task. EBs are composed of various cell types that express housekeeping genes at different levels [24]. Therefore, the analysis of ES cell differentiation in EBs is compounded by the fact that the gels with different stiffnesses induce proliferation of varying cell populations expressing housekeeping genes differently. Expression levels of common housekeeping genes HPRT, β tubulin and GAPDH significantly vary in EBs when compared on days 2 and 5 of differentiation, with GAPDH being the most stable out of all three [24]. As the duration of our experiment was much longer than 5 days, it is likely that the levels of the normalizer gene (GAPDH) considerably fluctuated at a time point when morphometric analysis was done. And finally, gene expression analysis may be less efficient for detection of less than 3-4-fold difference between conditions. Therefore, we propose that morphometric analysis may be more appropriate for analysis of the differentiation and cellular EB organization at advanced stages. Additionally, we suggest that PCR analysis reflected cardiomyogenic differentiation level of an entire EB group without regard for the amount of necrosis or histological finesse of EBs, while morphological analysis allowed us to assess both cellular composition and histological structure in individual EBs.

In the end, we envisage that the bioactive hydrogel matrix with proper stiffness will be useful for *in vitro* assembly and large-scale production of vascularized cardiac tissue that could be used for cell therapies of heart disease, *in vitro* organogenesis and toxicological assays [25]. The vascularized cardiac muscle tissue created *in vitro* may potentially be used as a graft for a focal strengthening of the heart wall. Additionally, this study will significantly assist efforts to rejuvenate patient's own regenerative potential of myocardium by elucidating the relationship between physiological hardening of heart muscle during development and gradual loss of proliferative capacity in cardiomyoblasts. Ultimately, our findings will bridge fundamental and applied aspects of cardiovascular development, pathology, and regeneration and take them to the next level.

## Conclusion

In conclusion, this study demonstrated *in vitro* creation of cardiovascular organoids by tailoring cardiomyogenic and endothelial differentiation of ES cells and intercellular organization in EBs with matrix rigidity, coupled with cell adhesion ligands. Specifically, collagen-conjugated hydrogels with stiffness similar to cardiac muscle tissue could promote the cell proliferation and co-differentiation to cardiomyocytes, compared to gels with stiffness of brain or non-mineralized bony tissue. Therefore, the EB-adherent hydrogel with similar stiffness to muscle could create a portion of heart-like tissue, in which striated muscle sheets are vascularized with branched blood vessels. The results of this study will be useful for better understanding of emergent behavior of EBs in developmental and regeneration process, extending application of EBs for drug screening, cell therapy, tissue regeneration and rejuvenation.

## Supporting Information

Figure S1 **Schematic describing preparation of the CCP gels.** Collagen-conjugated PEG acrylate was mixed with pre-gel solution of acrylamide, bis-acrylamide, and ammounium persulfate, in order to link collagen to polyacrylamide molecules in a hydrogel.
(TIF)

Figure S2 **SEM images of (A) the pure collagen gel with** ***E*** **of 0.2 kPa, (B) the CCP gel with** ***E*** **of 6 kPa and (C) the CCP gel with** ***E*** **of 40 kPa.** Gel disks were fixed, flash-frozen in liquid nitrogen, and desiccated for imaging.
(TIF)

Figure S3 **Cross-sectional images of EBs stained by Hematoxylin & Eosin.** EBs in (1) to (5) were cultured in medium supplemented with 10% FBS and those shown in (6) to (10) were cultured in FBS-free medium. (1 & 6) EBs cultured in a suspended state for 8 days. EBs were composed of tightly packed immature cells. Arrowheads indicate Reichert's membrane – thick basement membrane covered by endodermal cells on the external surface. (2 & 7) EBs cultured in suspended state for 23 days. (3 & 8) EBs cultured on the gel with an *E* of 0.2 kPa, (4 & 9) EBs cultured on the gel with *E* of 6 kPa, (5 & 10) EBs cultured on the gel with *E* of 40 kPa. In (2–4), Cystic EBs display large internal cavity (thin arrows) and outer surface lined by columnar epithelium (endoderm) (thick arrows). In (5), a flattened EB is composed of a multilayered sheet of tightly arranged cell with basophilic cytoplasm. In (7), the EB shows well-defined necrotic area in the center that contains cellular debris, with uniformly pink staining and lost nuclear details. The area of necrosis is circumscribed by a dotted line. In (8), the EB displays two neuroectodermal rosettes that are delineated by a dotted line. The inset in (9) contains a small outpouching that was exhibiting contractile activity. In (10), the EB shows an area of necrosis in the center. Scale bars represent 50 µm.
(TIF)

Figure S4 **FACS analysis of cardiomyogenic and endothelial differentiation in EBs.** EBs, cultured in suspension or on the gels were dissociated into individual cells, fixed and stained for sarcomeric alpha actinin and CD31. Cell agglomerates and cell doublets were excluded during the analysis. The plots reflect results of flow cytometry in EBs cultured in suspension (A), or on the gels with *E* of 0.2 (B), 6 kPa (C) and 40 kPa (D).
(TIF)

Figure S5 **Cell cycle analysis of non-cardiomyoblasts using EdU incorporation.** (A) Quantified percentage of the cells negative for SAA (non-cardiomyoblasts) incorporating EdU on Day 15 (A-1) and 23 (A-2). In (A-1), all differences in values are not statistically significant. In (A-2), the difference of values between EBs cultured on the gel with *E* of 0.2 kPa and EBs on the gels with *E* of 6 and 40 kPa was statistically significant (p<0.05). (C) The degree of decrease in the percentage of non-cardiomyoblasts incorporating EdU between Day 15 and Day 23.
(TIF)
