# Supplementary figures and images for: Matrix Rigidity-Modulated Cardiovascular Organoid Formation from Embryoid Bodies
Source: PLoS One. 2014 Apr 14;9(4):e94764. doi: 10.1371/journal.pone.0094764 (PMC3986240; doi:10.1371/journal.pone.0094764)

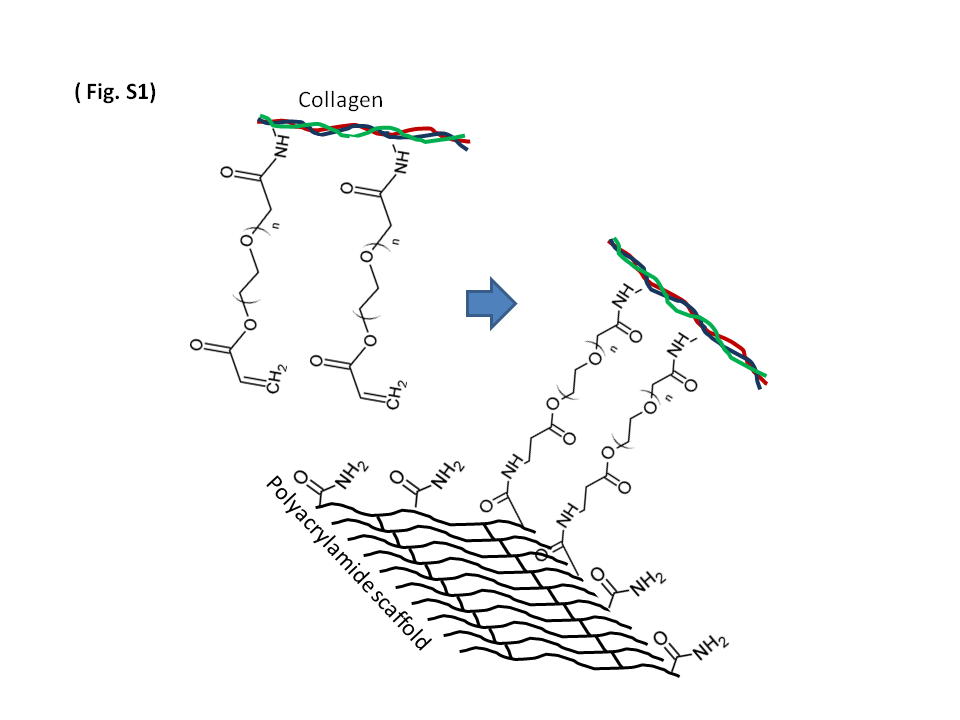

Supplement: Figure S1 — Schematic describing preparation of the CCP gels. Collagen-conjugated PEG acrylate was mixed with pre-gel solution of acrylamide, bis-acrylamide, and ammounium persulfate, in order to link collagen to polyacrylamide molecules in a hydrogel. (TIF) [file pone.0094764.s001.tif]

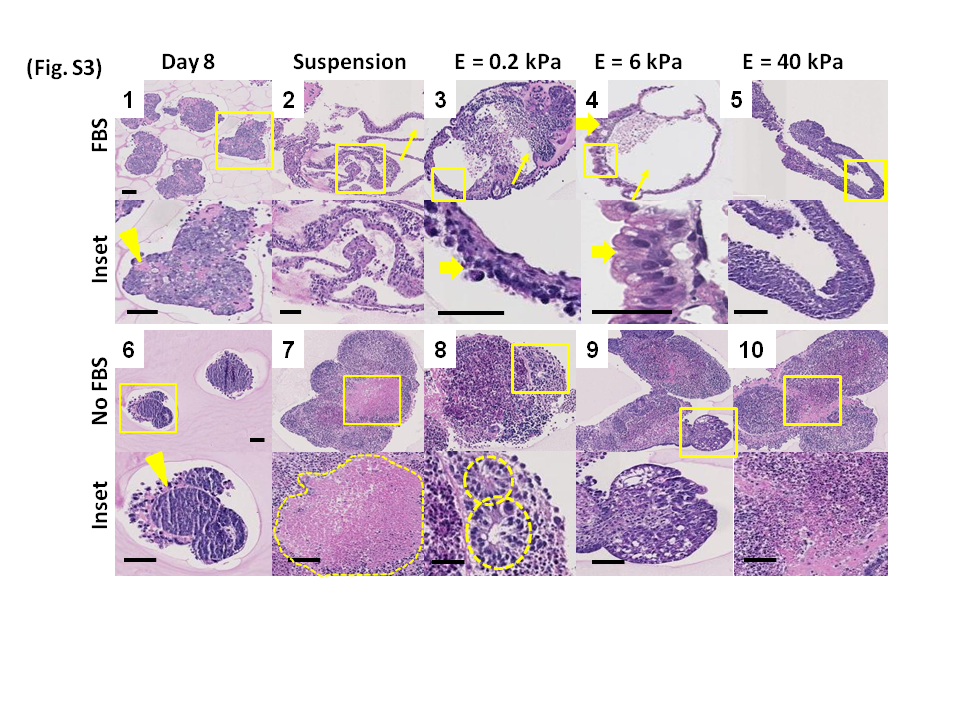

Supplement: Figure S3 — Cross-sectional images of EBs stained by Hematoxylin & Eosin. EBs in (1) to (5) were cultured in medium supplemented with 10% FBS and those shown in (6) to (10) were cultured in FBS-free medium. (1 & 6) EBs cultured in a suspended state for 8 days. EBs were composed of tightly packed immature cells. Arrowheads indicate Reichert's membrane – thick basement membrane covered by endodermal cells on the external surface. (2 & 7) EBs cultured in suspended state for 23 days. (3 & 8) EBs cultured on the gel with an E of 0.2 kPa, (4 & 9) EBs cultured on the gel with E of 6 kPa, (5 & 10) EBs cultured on the gel with E of 40 kPa. In (2–4), Cystic EBs display large internal cavity (thin arrows) and outer surface lined by columnar epithelium (endoderm) (thick arrows). In (5), a flattened EB is composed of a multilayered sheet of tightly arranged cell with basophilic cytoplasm. In (7), the EB shows well-defined necrotic area in the center that contains cellular debris, with uniformly pink staining and lost nuclear details. The area of necrosis is circumscribed by a dotted line. In (8), the EB displays two neuroectodermal rosettes that are delineated by a dotted line. The inset in (9) contains a small outpouching that was exhibiting contractile activity. In (10), the EB shows an area of necrosis in the center. Scale bars represent 50 µm. (TIF) [file pone.0094764.s003.tif]

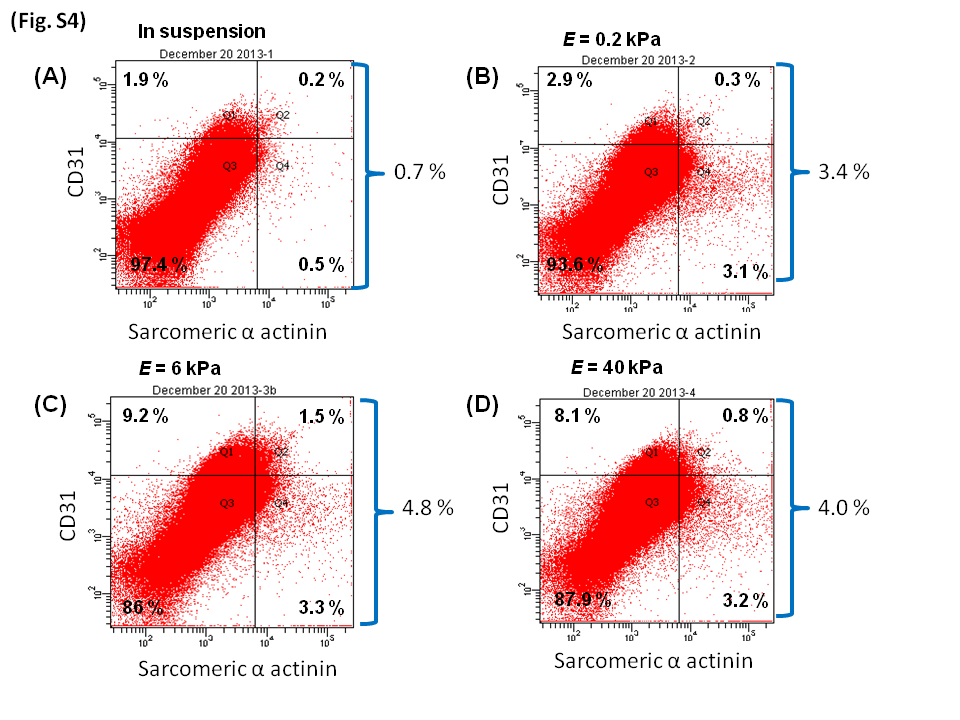

Supplement: Figure S4 — FACS analysis of cardiomyogenic and endothelial differentiation in EBs. EBs, cultured in suspension or on the gels were dissociated into individual cells, fixed and stained for sarcomeric alpha actinin and CD31. Cell agglomerates and cell doublets were excluded during the analysis. The plots reflect results of flow cytometry in EBs cultured in suspension (A), or on the gels with E of 0.2 (B), 6 kPa (C) and 40 kPa (D). (TIF) [file pone.0094764.s004.tif]
